# Hierarchical Porous RGO/PEDOT/PANI Hybrid for Planar/Linear Supercapacitor with Outstanding Flexibility and Stability

**DOI:** 10.1007/s40820-019-0342-5

**Published:** 2020-01-04

**Authors:** Fuwei Liu, Luoyuan Xie, Li Wang, Wei Chen, Wei Wei, Xian Chen, Shaojuan Luo, Lei Dong, Qilin Dai, Yang Huang, Lei Wang

**Affiliations:** 1 Shenzhen Key Laboratory of Polymer Science and Technology, College of Materials Science and Engineering, Shenzhen University, Shenzhen, 518060 People’s Republic of China; 2 College of Physics and Electronic Engineering, Xinyang Normal University, Xinyang, 464000 People’s Republic of China; 3 Institute of Medical Engineering, School of Basic Medical Sciences, Xi’an Jiaotong University, Xi’an, 710061 People’s Republic of China; 4 Key Laboratory of Advanced Technologies of Materials (Ministry of Education), School of Materials Science and Engineering, Southwest Jiaotong University, Chengdu, 610031 People’s Republic of China; 5 School of Chemical Engineering and Light Industry, Guangdong University of Technology, Guangzhou, 510006 People’s Republic of China; 6 Department of Physics, Southern University of Science and Technology, Shenzhen, 518055 People’s Republic of China; 7 Department of Chemistry, Physics, and Atmospheric Sciences, Jackson State University, Jackson, MS 39217 USA

**Keywords:** RGO/PEDOT/PANI, Hierarchical, Porous, Supercapacitor, High performance

## Abstract

Many hybrid electrodes for supercapacitors (SCs) are a reckless combination without proper structural design that keeps them from fulfilling their potential. Herein, we design a reduced graphene oxide/poly(3,4-ethylenedioxythiophene)/polyaniline (RGO/PEDOT/PANI) hybrid with hierarchical and porous structure for high-performance SCs, where components fully harness their advantages, forming an interconnected and conductive framework with substantial reactive sites.Thus, this hybrid achieves a high capacitance of 535 F g^−1^ along with good rate capability and cyclability. The planar SC based on this hybrid deliver an energy density of 26.89 Wh kg^−1^ at a power density of 800 W kg^−1^. The linear SC developed via modifying a cotton yarn with the hybrid exhibits good flexibility and structural stability, which operates normally after arbitrary deformations. This work provides a beneficial reference for developing SCs.

**Electronic supplementary material:**

The online version of this article (10.1007/s40820-019-0342-5) contains supplementary material, which is available to authorized users.

## Highlights

- Hierarchical porous reduced graphene oxide/poly(3,4-ethylenedioxythiophene)/polyaniline hybrid was designed.
- The hybrid achieves a high capacitance of 535 F g^−1^ along with a good rate capability and cyclability.

**Electronic supplementary material:**

The online version of this article (10.1007/s40820-019-0342-5) contains supplementary material, which is available to authorized users.

## Graphical abstract

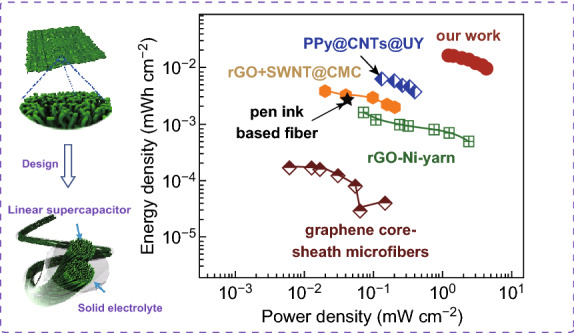

## Introduction

Supercapacitor (SC), with high power density, fast charge/discharge rate, good safety, and outstanding cycling stability, is a promising energy storage device that can bridge the gap between traditional electrolytic capacitor and batteries and can be applied to a variety of fields, such as hybrid trucks/buses, uninterruptible power supplies, and load-leveling systems for intermittent energy system [1–3]. However, compared with familiar batteries, SCs normally have relatively low energy storage capability (e.g., energy density), thus limiting its widespread application in high energy-consuming devices (e.g., mobile phone) [3, 4]. To date, extensive efforts have been made to solve this challenging problem, ranging from the application of novel active materials to the introduction of fancy structures [4–6].

Exploring an ideal active material with intrinsic high performance for SCs should be the most straightforward way. Apart from traditional carbon materials, metal oxides, conductive polymers, and some non-conductive polymers with electrochemically active sites [7–9], many distinctive new materials have been synthesized and applied to fabricate SCs recently. For example, a number of two-dimensional (2D) materials, such as graphene [10], MXene [11, 12], antimonene [13], phosphorene [14], were reported to show high capacitance for SCs due to their large surface area, attached functional groups, moderate interlayer space, and/or intrinsic high conductivity. Nevertheless, the intrinsic characteristics of these 2D materials can cause certain obstacles in designing high-performance SCs. For example, these tiny 2D sheets might accumulate together compactly when assembled into an electrode that impedes the electrolyte and ion penetration, leading to the capacitance lower than theoretical value [15]. Besides, some 2D materials are less conductive or with unstable structure (e.g., phosphorene) that also leads to unsatisfied capacitive performance.

To exert the positive effects/advantages of different active materials, one promising solution is to selectively combine them as a hybrid system that can effectively nullify their respective disadvantages and fully release the potentiality. For example, through making a hybrid with polypyrrole (PPy), the non-conductive phosphorene becomes conductive due to the coating of conductive polymer, bringing about fast ion and electron transport, thus achieving high capacitance; in the meantime, cycling performance of PPy is enhanced owing to the buffer effect of these flexible 2D sheets [16]. Similarly, by combining low-conductive transition metal hydroxides/oxides with carbon nanomaterials, the as-prepared hybrid electrode exhibits good conductivity and abundant electrochemically active sites that benefit the capacity and cycling stability [17, 18]. Obviously, the hybrids between different active materials should have enormous development space for high-performance SCs, since there are so many existed traditional materials together with the emerging new materials.

However, only with a reasonable combining structure, there will be a synergistic effect in the hybrid that can maximize the positive roles of each component, bringing about better capacitance, cycling stability, and rate capability. Or else capacitive performance of the complex hybrid can be a simple sum of individual components; in an extreme case, its capacitance might be even less than the value contributed by one single component. Thus, an excellent structural design is indispensable when fabricating high-performance SCs based on hybrid materials. However, it is challenging to achieve such a special structure that coordinates the relationship between each component, considering every component in the complex has its own characteristics, which sometimes might even contradict with each other.

Herein, we demonstrate the design of a flexible and lightweight reduced graphene oxide/poly(3,4-ethylenedioxythiophene)/polyaniline (RGO/PEDOT/PANI) hybrid with hierarchical and porous structure for high-performance SCs. Each component in the hybrid fully harnesses its own advantages while forming an interconnected conductive framework with substantial interfaces/reactive sites for reversible electrochemical reaction, which results in a high capacitance of 535 F g^−1^ together with good rate capability and cycling stability. This hybrid was further applied to fabricate a planar SC, which delivered an energy density of 26.89 Wh kg^−1^ at a power density of 800 W kg^−1^. In addition, we succeeded in developing a linear SC by modifying a non-conductive cotton yarn with this hybrid. The great flexibility and structural stability endowed this linear prototype with outstanding practicability, which powered a digital watch continuously when it was stretched, bended, twisted, or knotted arbitrarily. Three of them connected in series easily powered this watch for over half an hour. We believe this hybrid can inspire the designed combination of different active materials.

## Results and Discussion

### Design of Hierarchical and Porous RGO/PEDOT/PANI Hybrid

**Fig. 1:**
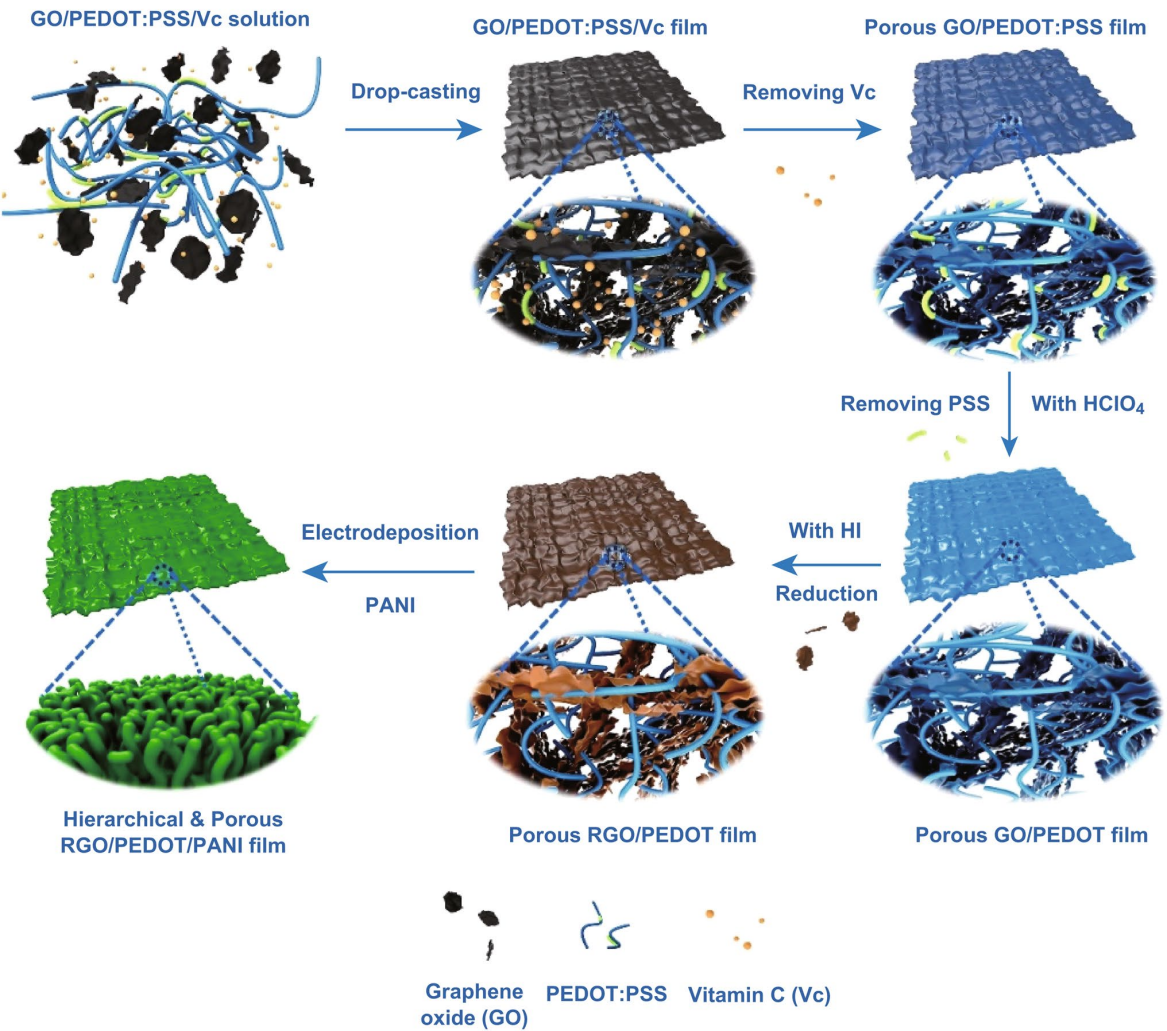
Fabrication process of hierarchical and porous RGO/PEDOT/PANI hybrid

The design of hierarchical and porous RGO/PEDOT/PANI hybrid is presented in Fig. 1. The graphene oxide (GO), poly(3,4-ethylenedioxythiophene):poly(styrenesulfonate) (PEDOT:PSS), and vitamin C (Vc) are mixed together to form a dark blue solution that can be applied to prepare a GO/PEDOT:PSS self-standing hybrid via a facile dropped casting strategy. To solve the restacking of GO, Vc, the spacer and pore-forming agent, is removed by soaking this hybrid in water, simultaneously creating a porous structure that is beneficial for electrolyte penetration and increases surface area simultaneously. As intended, integrity of the interconnected framework in this porous hybrid is well reserved, because the GO nanosheets are still glued together by PEDOT:PSS, which is not achieved in some porous hybrids [17, 18]. With a subsequent treatment of perchloric acid (HClO_4_), conductivity of PEDOT:PSS is greatly enhanced due to the removal of PSS and the formation of ordered structure. In addition, we further improve the electrical conductivity of this porous framework via a secondary acid treatment by hydroiodic acid (HI), a reduction agent for GO that can eliminate the attached oxygen-containing functional groups and restore the corresponding structural defects [19]. Thanks to such a high conductivity, PANI nanorods with exceptional pseudo-capacitance are electrodeposited all over the hybrid easily and consequently forming a hierarchical and porous structure. Compared with some representative inorganic hybrids, the RGO/PEDOT/PANI is fabricated mainly based on lightweight organic materials via a simple and convenient method that has high practicality [17, 18]. Moreover, the RGO/PEDOT/PANI should exhibit better electrochemical properties compared with a number of hybrid electrodes, since its well-designed hierarchical and porous structure brings several advantages as follows: (1) The interconnected conductive framework ensures fast electron and ion transport, (2) the porous structure facilitates electrolyte penetration and increases surface area, (3) the hierarchical structure provides substantial interfaces/reactive sites for electrochemical reactions without destroying the porosity, (4) the buffering effect of flexible framework benefits stability during long-term cycles and repeated deformations, etc. Besides its electrochemical performance, the self-standing RGO/PEDOT/PANI has bright prospect in constructing flexible SCs for wearable devices, which is not easily achieved by the powdery hybrids.

### Optimization of Hierarchical and Porous Structure

The hierarchical and porous structure of RGO/PEDOT/PANI affects its electrochemical performance. Thus, a series of composition and structure optimizations were carried out to fully develop its potentiality. GO was chosen as the main skeleton, because of its large surface area and good flexibility, which not only provides certain capacitance but also enhances structural stability under long-term cycles. By using PEDOT:PSS as a glue, an interconnected network is formed between these spontaneously stacked GO sheets so that they have enhanced integrity. After subsequent acid treatments by HClO_4_ and HI, PSS was removed from PEDOT:PSS and GO was reduced into RGO, resulting in a conductive and interconnected RGO/PEDOT framework for the effective ion and electron transport. The highest capacitance of RGO/PEDOT was achieved when the mass percentage of RGO was 70%, offering a value of 136 F g^−1^ (Fig. S1). The electrochemical performance of both components in the framework is maximized with such a proportion. Because of lower or higher percentage of RGO, surface area and conductivity of RGO/PEDOT are affected and reduced with a certain degree, which causes poor electrochemical activity and thus a decreased capacitance as shown in the cyclic voltammogram (CV) and galvanostatic charge-discharge (CD) files of Fig. S1.

**Fig. 2:**
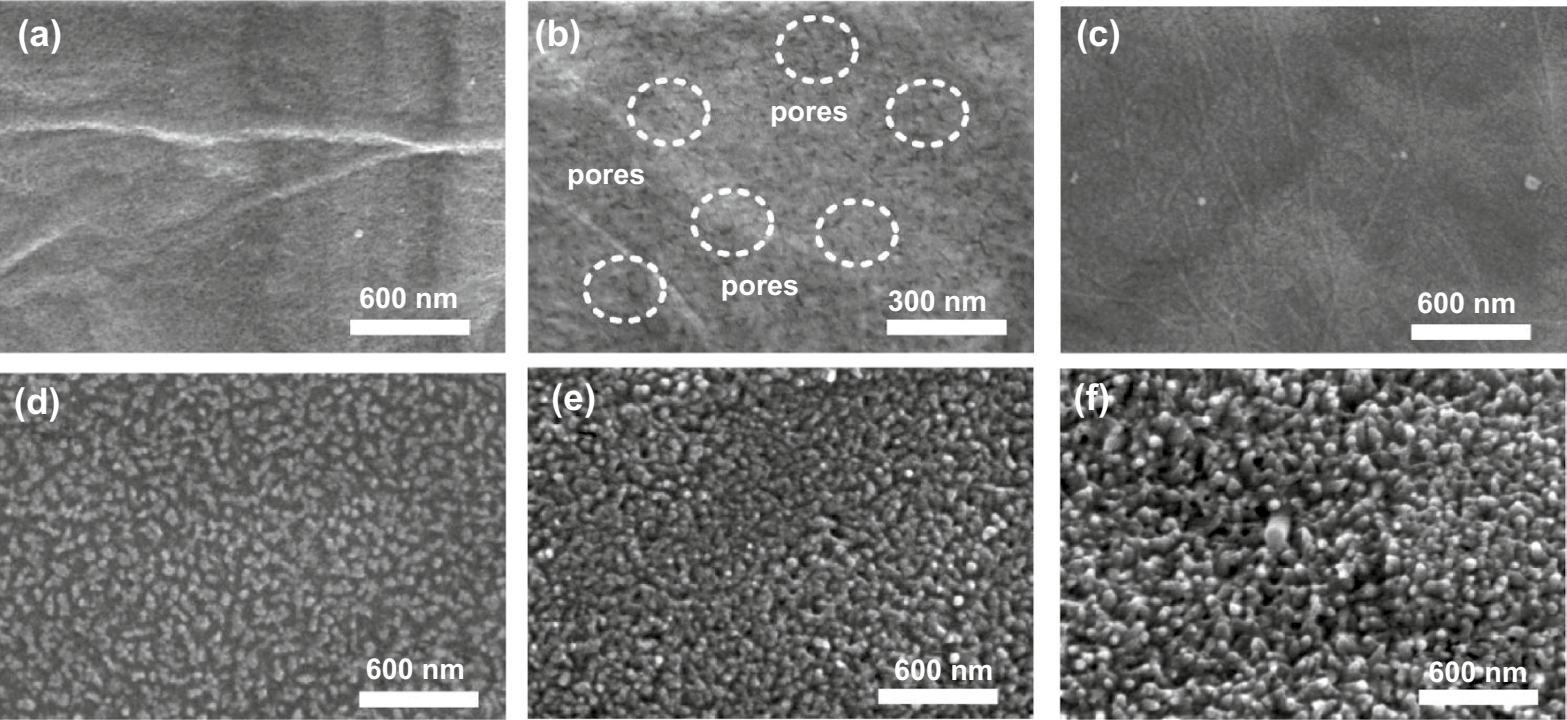
**a**, **b** Scanning electron microscopy (SEM) images of porous RGO/PEDOT framework at different scales, **c**–**f** SEM of hierarchical and porous RGO/PEDOT/PANI hybrid with different electrodeposition durations of PANI: 5, 10, 15, and 20 min

Without an intentional pore-forming strategy, the RGO/PEDOT framework only presents a dense surface and a compact cross section, in which PEDOT intimately covers on the RGO without affecting its 2D morphology (Fig. S2). This is unfavorable for effective electrochemical reaction, since electrolyte cannot penetrate into the framework. Unsurprisingly, capacitance of the rigid RGO/PEDOT is far from satisfactory (Fig. S3a, b). To solve this challenge, we introduced porous structure both on the surface and in the body of RGO/PEDOT (Figs. 2a, b and S4a–c), which assists the penetration of electrolyte, leading to an obvious improvement in capacitance. By adding Vc with the optimum value (~ 600 wt%), the capacitance of pristine RGO/PEDOT was nearly doubled to 228 F g^−1^ (Fig. S3c), while the gradually obvious oxidation and deoxidation peaks in CV curves evidently proved the porous structure facilitated the electrochemical reaction of RGO/PEDOT effectively (Fig. S3a). Besides, due to its conductivity and flexibility, the porous RGO/PEDOT exhibited outstanding rate capability and cycling stability, which not only provided 79.6% of capacitance (181.5 F g^−1^) at a high current density of 15 A g^−1^, but also realized 99.8% capacitance retention over 10,000 cycles (Fig. S3d–f). However, once addition of Vc is over the optimum condition, integrity of RGO/PEDOT will be affected obviously, since macroscopic pores instead of microscopic ones appear all over its surface (Fig. S5). Surely, these large pores deteriorate the interconnection of conductive framework and cause the performance degradation to a certain degree; for example, the capacitance decreased to 221 F g^−1^ when the addition of Vc increased to 680 wt%.

To further promote the capacitance, PANI nanorods with intrinsically high capacitance were electrodeposited on the whole body of porous RGO/PEDOT framework (Figs. 2c–f and S4) and consequently forming a hierarchical structure with substantial interfaces/reactive sites for the electrochemical reactions. The resultant RGO/PEDOT/PANI hybrid achieves a significant high capacitance of 535 F g^−1^ with the optimum electrodeposition duration of PANI (Fig. S6), which is comparable with or even better than that of other related hybrid electrodes, as shown in Table S1 in detail. It is noteworthy that the specific capacitance of PANI nanorods based on its own weight is around 953 F g^−1^, which exploits its inherent capacitance effectively [20–22], one important reason for the increased capacitance of our hybrid. When with a shorter electrodeposition duration, the PANI nanorods are not grown sufficiently on the porous framework (Fig. 2c, d), leading to insufficient interfaces/reactive sites and thus a decreased capacitance, whereas with a longer electrodeposition duration, the PANI nanorods are grown excessively that blocks the porous structure, impeding the penetration of electrolyte and the rapid transport of electron/ion, which is unfavorable for activating this hybrid and results in a reduced capacitance (Fig. S6). In addition, the electrical conductivity of RGO/PEDOT framework is not obviously affected even after PANI electrodeposition, since system resistance (*R*_s_) stays almost unchanged as shown in the inset of Fig. S6c. This is another reason for the outstanding performance of this hierarchical and porous hybrid.

### Outstanding Performance and Mechanism Behind

**Fig. 3:**
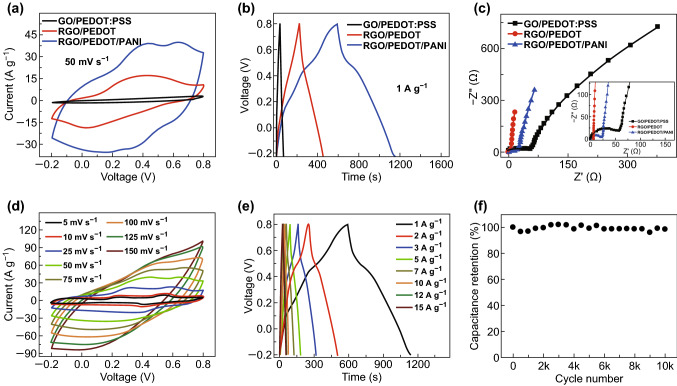
**a** CV curves of GO/PEDOT:PSS, RGO/PEDOT, and RGO/PEDOT/PANI at 50 mV s^−1^, **b** CD curves of GO/PEDOT:PSS, RGO/PEDOT, and RGO/PEDOT/PANI at 1 A g^−1^, **c** Nyquist plots of GO/PEDOT:PSS, RGO/PEDOT, and RGO/PEDOT/PANI, **d** CV curves of RGO/PEDOT/PANI at different scan rates, **e** CD curves of RGO/PEDOT/PANI at different current densities, and **f** cycle stabilities of RGO/PEDOT/PANI hybrid at 5 A g^−1^

Based on the hierarchical and porous design, the RGO/PEDOT provides conductive and interconnected framework for fast electrochemical reactions, while PANI nanorods offer enough reactive sites to improve capacitance. The synergistic effect in the hybrid brings about outstanding electrochemical performance, and the enclosed area of corresponding CV curves increases successively and substantially (Fig. 3a). Similarly, the CD curves confirm the advantages of hierarchical and porous structure, because RGO/PEDOT/PANI exhibited longer discharge period under the same current density compared with GO/PEDOT:PSS and RGO/PEDOT (Fig. 3b). Thanks to the acid treatments, charge transfer resistance (*R*_ct_) of RGO/PEDOT decreased significantly, which slightly increased after PANI electrodeposition (Fig. 3c). This good conductivity ensures rapid electrochemical reaction at the interfaces/reactive sites between electrode and electrolyte. Thus, RGO/PEDOT/PANI shows rapid response in the CV curves with reversible redox peak couples under different scan rates, corresponding to the transition between leucoemeraldine and protonated emeraldine, and the Faradaic transformation between emeraldine and pernigraniline, respectively (Fig. 3d) [23]. Moreover, due to the low *R*_s_ and *R*_ct_ (Figs. 3c and S7a), CD curves maintained symmetric quasi-triangular shape even under high current densities (Fig. 3e), which provided a high capacitance of 388.5 F g^−1^ at 15 A g^−1^ (Fig. S7b), showing a good rate capability. Because the main skeleton is the flexible RGO/PEDOT that has outstanding cyclability (Fig. S3f), RGO/PEDOT/PANI exhibits similar good structural stability under long-term cycles, which provides over 99% capacitance retention after 10,000 charge/discharge cycles (Fig. 3f).

**Fig. 4:**
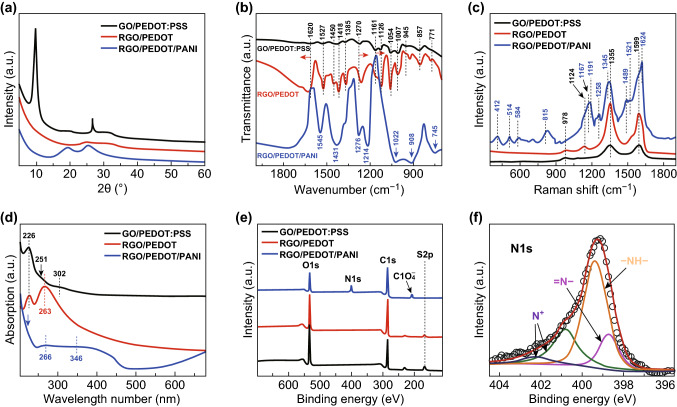
**a** XRD patterns, **b** FTIR spectra, **c** Raman spectra, **d** UV–Vis spectra, **e** XPS full spectra of GO/PEDOT:PSS, RGO/PEDOT, and RGO/PEDOT/PANI, and **f** N 1*s* spectra of RGO/PEDOT/PANI

The outstanding electrochemical performance of RGO/PEDOT/PANI is mostly attributed to the designed structure. X-ray diffraction (XRD) is firstly employed to reveal its atomic and molecular structures (Fig. 4a). The sharp diffraction peak at 9.7° in GO/PEDOT:PSS is mainly attributed to the (002) facet of GO. After acid treatments, there is only an inconspicuous peak located at 24.9° in RGO/PEDOT, initiated by the reduction in GO [24]. Actually, molecular structure of PEDOT:PSS changes after acid treatment as well, which is not reflected in Fig. 4a, because of its relatively weak diffraction peak. As shown in Fig. S8a, PEDOT:PSS only shows a peak with low intensity at 26.4° assigned to the interchain planar ring stacking of PEDOT [25, 26]. Due to the shielding of GO, this peak is inconspicuous in GO/PEDOT:PSS. After acid treatment, one new diffraction peak appears at 18.5° related to the amorphous halo of PSS, suggesting an improved crystallinity of PSS [25–27]. Meanwhile, the diffraction peak intensity related to the (010) facet of PEDOT at 26.4° increases obviously, another proof of improved crystallinity [25–27]. These results indicate that acid treatment induces the rearrangement of PEDOT:PSS, leading to an ordered lamellar stacking and continuous network. This crystalline order structure in conducting polymers facilitates efficient intra- and interchain charge transport, resulting in a high conductivity [27, 28]. Therefore, the acid treatments promote the formation of a conductive and continuous network in RGO/PEDOT. After PANI electrodeposition, two broad peaks centered at 19.2° and 25.3° are almost the same with pure PANI (Fig. S8b), which are assigned to the repeat units that are periodically perpendicular and parallel to PANI backbone chains [29]. Noticeably, comparing with pure PANI, both characteristic peaks become sharper, suggesting an improved ordering degree of PANI while with RGO/PEDOT, which brings about good carrier mobility in RGO/PEDOT/PANI.

In addition to XRD, Fourier-transform infrared spectroscopy (FTIR) reveals the chemical bonding or molecular structure of RGO/PEDOT/PANI. For GO/PEDOT:PSS (Fig. 4b), its peaks associated with C–O–C bonds and C–S bonds slightly shift to 1270 and 857 cm^−1^, as compared with pristine PEDOT:PSS (Fig. S9a), resulting from the strong interactions between GO and PEDOT:PSS [30, 31]. As for RGO/PEDOT, variation in characteristic peaks is mainly induced by GO reduction (Fig. S9). Besides, the peak belonging to S–O of PSS in PEDOT:PSS at 1161 cm^−1^ decreases and become a little shoulder peak, indicating the removal of PSS after acid treatment [30]. After electrodeposition, a series of typical peaks corresponding to the doped PANI appears in the hybrid as shown in Table S2 [23, 32]. Additionally, some peaks shift to lower wave number compared with pure PANI (Fig. S9d). These results suggest that PANI nanorods were firmly grown on RGO/PEDOT with strong interfacial interactions, which is possibly attributed to the hydrogen bonding and π–π interaction [33]. Therefore, electron and ion transport through this hybrid effectively during electrochemical reaction, leading to the outstanding performance as shown in Fig. 3.

As a supplement to FTIR, Raman spectroscopy also underlines the structural variation in GO/PEDOT/PANI. The GO/PEDOT:PSS shows three main characteristic peaks at 978, 1355, and 1599 cm^−1^ (Fig. 4c), which are assigned to oxyethylene ring deformation of PEDOT:PSS, D and G bands of GO, respectively [34–36]. After acid treatments, intensity of D and G bands increases drastically. However, there is no obvious Raman shift in pure PEDOT:PSS (Fig. S10), indicating that the increased intensity of D and G bands is mainly initiated by the structural change of GO and/or the strong interactions between RGO and PEDOT. Usually, defect density of graphitic structure is evaluated by the intensity ratio between D and G bands (*I*_D_/*I*_G_) [36, 37]. After acid treatments, the corresponding value of *I*_D_/*I*_G_ in GO/PEDOT:PSS slightly increases from 0.91 to 1.06, which is apparently lower than pure RGO (1.87, Fig. S10). The improvement is due to PEDOT introduction that fills the holes and/or voids generated by GO reduction [36]. Besides, as a fingerprint of RGO, the typical peak of C–C interring bending variation appears at 1124 cm^−1^. The strong interactions between RGO and PEDOT facilitate the charge transport between the components, which is consistent with previous characterizations.

Whereas the Raman spectra of RGO/PEDOT/PANI is much more different from its predecessors of GO/PEDOT:PSS and RGO/PEDOT (Fig. 4c). In addition to D and G bands of GO, some new peaks belong to PANI emerge as shown in Table S3 [38, 39]. The C–N^+^ vibration (quinoid ring) peak slightly shifts to higher wave number direction (1345 cm^−1^) compared with pure PANI (Fig. S10c), while N–H stretching vibration peak shifts to lower wave number direction (1521 cm^−1^) with much higher intensity. These results indicate the intimate contact between PANI and RGO/PEDOT, benefitting electron transport between the interfaces of hierarchical structure and leading to a better performance. In addition, RGO/PEDOT/PANI exhibits higher band intensity of emeraldine salt form (1345 cm^−1^) than pure PANI, indicating that the PANI remains at highly doped state, which makes substantial contribution to the capacitance as shown in Fig. 3.

Conformational and compositional changes of RGO/PEDOT/PANI are confirmed by UV–Vis spectroscopy as well. The related spectrum reveals the PSS decrement in PEDOT:PSS and GO reduction after acid treatments (Figs. 4d and S11a, b) [40, 41]. Besides, RGO/PEDOT presents different absorption peaks with PEDOT and/or GO (Fig. S11a–c), which confirms the strong interaction between PEDOT and RGO, resulting in good ion/electron transportation. After PANI electrodeposition, the hybrid shows absorbance peaks at 266, 346, and 769 nm, assigned to *π*–π* and polaron–π*, and *π*–polaron transitions, respectively (Figs. 4d and S11d) [41, 42]. All these peaks are the typical characteristics of conductive PANI in highly doped state [41, 42]. When compared with pure PANI, peaks of *π*–π* and polaron–π* in RGO/PEDOT/PANI were blue-shifted (Fig. S11d), together with the evident increase in peak intensity, suggesting the strong interactions between PANI and RGO/PEDOT. Meanwhile, *π*–polaron transitions appear as an apparent peak with high intensity, whereas in pure PANI it only shows a platform with free tail extended to IR region, indicating that PANI was grown on RGO/PEDOT with an increased conjugated degree (Fig. S11d). Therefore, electron and ion pass through the highly doped PANI effectively, promoting the fast redox reactions as shown in Fig. 3.

The surface chemistry of RGO/PEDOT/PANI related to its structural advantages was further investigated by XPS. The C, N, O, and S are presented in the full spectra (Fig. 4e). After acid treatments, the peak related to S decreases obviously due to the selective removal of PSS from PEDOT:PSS [27, 43], which is confirmed by the changes of S 2*p* peaks in pure PEDOT:PSS with identical acid treatments (Fig. S12) [43–45]. The decrease in PSS not only assists the structural rearrangement of PEDOT, but also improves the concentration of charge carriers, increasing the conductivity evidently. The intensity of N 1*s* arises obviously after PANI electrodeposition (Fig. 4e). Deconvolution of N 1*s* shows four peaks positioned at 398.7, 399.4, 400.8, and 402.2 eV, corresponding to quinoid imine, benzenoid imine, positively charged imine, and protonated amine, respectively (Fig. 4f). The abundant quinoid imine (–N=) enhances the electron delocalization, ensuring high conductivity of PANI chains. [46–48] Besides, the peak appearing at 208 eV in the full spectrum is ascribed to an enriched doping of ClO_4_^−^ in PANI, which results in numerous unsaturated bonds and provides substantial reactive sites for the redox reaction [49]. Accordingly, RGO/PEDOT/PANI exhibits a higher capacitance (535 F g^−1^) compared with RGO/PEDOT (228 F g^−1^).

### Application in Flexible Supercapacitor Devices

**Fig. 5:**
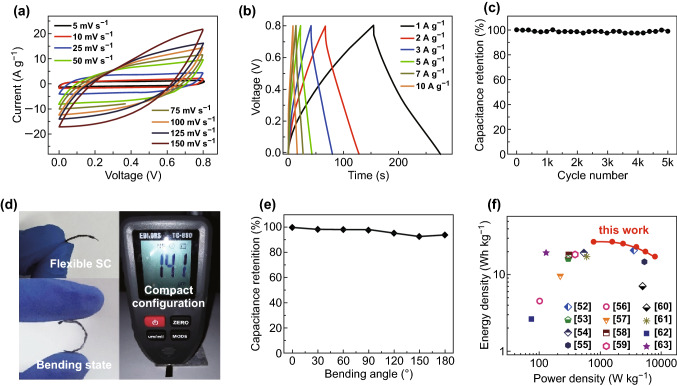
**a** CV curves of planar solid-state SC at different scan rates, **b** CD curves of the planar device at different current densities, **c** cycle stabilities of the device at 5 A g^−1^, **d** photographs of the compact and flexible SC, with a thickness of 141 μm, **e** capacitance retention at different bending angles, and **f** Ragone plots of our SC device and its comparison with other devices: Cr_2_O_3_/GO/PANI [52], PANI NFs/FrGO [53], RGO/CuO/PANI [54], CNT/PANI [55], CNF/PANI [56], HPC/PANI [57], PANI/MWCNT [58], RGO/MnO_2_@PANI [59], grapheme//MGC [60], activated carbon//MnO_2_ [61], MnO-RGO//FC [62], and activated carbon//NaMnO_2_ [63]

The hierarchical and porous RGO/PEDOT/PANI hybrid possesses outstanding performance together with good flexibility, making it a promising electrode for flexible SC. As shown in Fig. 5a, CV curves of the as-assembled SC remained quasi-rectangular shapes under a variety of scan rates, indicating a good reversibility of electrochemical reaction. Likewise, the triangular CD curves exhibited nearly symmetric shapes with different current densities (Fig. 5b), suggesting the smooth proceeding of electrochemical reaction during charge/discharge. With a current density of 1 A g^−1^, this SC device provides a high capacitance of 302.5 F g^−1^ that is comparable or even better than a number of reported devices based on different electrodes, such as PANI-GO (143.2 F g^−1^) [50], carbon nanofibers@PPy@graphene (188 F g^−1^) [51], Cr_2_O_3_/GO/PANI and Cr_2_O_3_/GO/PPy composites (263 and 100 F g^−1^) [52], PANI/functionalized RGO (324.4 F g^−1^) [53], and RGO-copper oxide-PANI (213.2 F g^−1^) [54]. Because RGO/PEDOT/PANI has an outstanding cycling stability, the SC possesses similar good structural stability, which presents limited capacitance fading after 5000 cycles (Fig. 5c). Owing to the great flexibility of hybrid, we can easily fabricate a highly flexible SC with a compact configuration (Fig. 5d), which is readily bent into arbitrary angle without degrading its capacitance apparently (Figs. 5e and S13). Energy density and power density are two parameters that can help to determine the energy storage performance of SCs. The SC with our hierarchical and porous hybrid achieves a high energy density of 26.89 Wh kg^−1^ at a power density of 800 W kg^−1^, showing an excellent energy storage capability. As shown in the Ragone plots (Fig. 5f), this performance is better than a few recently reported SCs [52–63].

**Fig. 6:**
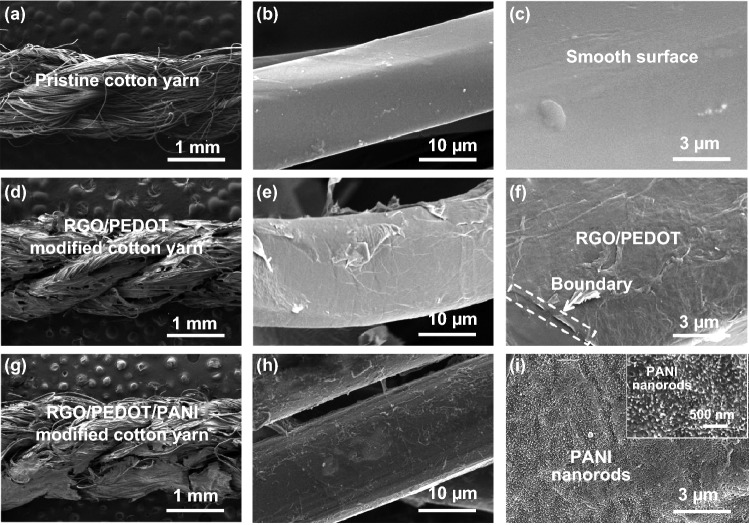
SEM images of the cotton electrode at different scales: **a**–**c** pristine state, **d**–**f** modified with RGO/PEDOT, and **g**–**i** modified with RGO/PEDOT/PANI hybrid

**Fig. 7:**
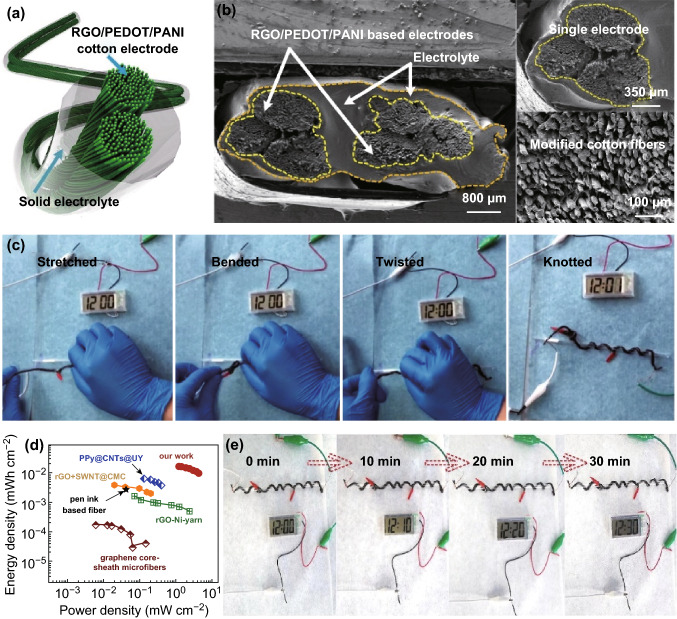
**a** Schematic diagram of the linear SC device, **b** cross-sectional SEM images of the linear device based on RGO/PEDOT/PANI-modified cotton yarn, **c** the linear device can be stretched, bended, twisted, or knotted arbitrarily without damaging its integrity, while continuously powering a digital watch, **d** Ragone plots of our linear SC and its comparison with other linear devices, and **e** three SCs connected in series can easily power a digital watch for at least half an hour

Other than planar type, linear prototype is another important configuration for flexible SC, which provides substantial flexibility and meets with knitting/weaving technology in textiles industry. Through processing the linear substrate with a series of modifications of dip-coating, acidic treatments and electrodeposition, we fabricate a linear SC based on RGO/PEDOT/PANI. It is to note that we choose a non-conductive cotton yarn as the substrate for linear SC, which is converted into a highly conductive one after dip-coating and acidic treatments (Fig. S14). This clearly demonstrates the strong applicability and generality of our modification strategy in fabricating linear SC with different yarns disregarding their intrinsic conductivity. As shown in the SEM images, the cotton yarn is consisted of numerous fibers with a diameter of ~ 13 μm (Fig. 6a, b). Before dip-coating of GO/PEDOT:PSS, these pristine fibers have very smooth surface (Fig. 6b, c); after coating GO/PEDOT:PSS and subsequent acidic treatments, these fibers are wrapped by a conductive RGO/PEDOT layer, exhibiting a rough surface with obvious wrinkles (Fig. 6d–f). On account of an intimate contact between the fibers and RGO/PEDOT, a highly conductive network is constructed across the cotton yarn continuously and spontaneously, leading to a low resistance as shown in Fig. S14. Afterward, PANI is readily electrodeposited on the RGO/PEDOT, thus forming the RGO/PEDOT/PANI hybrid alike the planar electrode (Fig. 6g–i). Finally, two of these as-modified cotton electrodes in parallel are assembled into a linear SC by wrapping with a suitable amount of solid electrolyte (Fig. 7a). The cross-sectional SEM images show that the solid electrolyte can penetrate into the gap between those tiny fibers of the electrode thoroughly (Fig. 7b), ensuring the activation of electrode materials and providing substantial interfaces for electrochemical reaction. Accordingly, the linear SC exhibits a decent capacitance alike the planar prototype. The CV and CD files show quasi-rectangular and -triangular shapes under a variety of scan rates and current densities, indicating ideal capacitive behavior accompanied with rapid and reversible electrochemical reactions (Fig. S15a, b). This linear SC provides a high capacitance of 179.5 mF cm^−2^, which is better than many other linear SCs, such as all-graphene core–sheath microfiber SC (1.2–1.7 mF cm^−2^) [64], RGO-Ni-yarn SC (72.1 mF cm^−2^) [65], fiber SC based on pen ink (11.9–19.5 mF cm^−2^) [66], coaxial wet-spun yarn SC based on RGO + CNT@CMC (177 mF cm^−2^) [67], PPy@CNTs@UY yarn SC (69 mF cm^−2^) [68], CNT fiber-based wire-shaped SC (4.63–4.99 mF cm^−2^) [69], and SC based on CNTs and ordered mesoporous carbon (39.7 mF cm^−2^) [70].

One strong advantage of linear SC is its substantial flexibility. As for our linear prototype, thanks to the intimate contact between electrode and electrolyte (Fig. 7b), it not only presents high flexibility, but also possesses good stability. Actually, its capacitance was not affected when the SC was bended from 0^°^ to 180^°^ (Fig. S16), which proves its great flexibility and structural stability under deformation. Moreover, it can be stretched, twisted, or knotted arbitrarily without damaging the structural integrity and thus powers a digital watch without any interruption as shown in Video S1 and Fig. 7c. In addition to the above advantages, this linear SC has favorable energy storage capability, achieving an energy density of 0.016 mWh cm^−2^ with a power density of 1.212 mW cm^−2^, which is comparable with or even better than several advanced linear SCs (Fig. 7d) [64–68]. Because of such good performance, the SCs can be connected in series or in parallel to meet different needs; for example, three SCs connected in series can easily power a digital watch for at least half an hour as demonstrated in Video S2 and Fig. 7e. This illustrates the practicability of our linear SC for different flexible electronics.

## Conclusions

In summary, we have successfully designed and fabricated a RGO/PEDOT/PANI hybrid with hierarchical and porous structure that can be directly used as flexible electrode for high-performance SCs. Porous structure was firstly introduced into the GO/PEDOT:PSS framework by removing embedded pore-forming agent, which facilitates electrolyte penetration. Afterward, this porous GO/PEDOT:PSS framework was treated with two different acids to improve its electrical conductivity, which not only removed the PSS in PEDOT:PSS and assists its realignment, but also reduced the GO to RGO. Thus, PANI nanorods can be electrodeposited on the highly conductive RGO/PEDOT framework and formed a flexible hybrid with hierarchical and porous structure, providing substantial interfaces/reactive sites for reversible electrochemical reactions. As convinced by the characterization results, each component of this hybrid can fulfill its potential, leading to a high capacitance (535 F g^−1^), a good rate capability (388.5 F g^−1^ at 15 A g^−1^), and an outstanding cyclability (99% capacitance retention over 10,000 cycles). The planar SCs based on the self-standing hybrid exhibited satisfactory performance, delivering an energy density of 26.89 Wh kg^−1^ at a power density of 800 W kg^−1^. Furthermore, we developed a linear SC through modifying the non-conductive cotton yarn with this hybrid, which presented remarkable flexibility, structural stability, and energy storage capability. This work paves an effective way to improve the performance of hybrid materials for flexible SCs via the structural design.

## Electronic supplementary material

Below is the link to the electronic supplementary material.

Supplementary material 1 (PDF 1507 kb)

Supplementary material 2 (AVI 3950 kb)

Supplementary material 3 (AVI 6040 kb)
