# Supplementary material for: Hierarchical Porous RGO/PEDOT/PANI Hybrid for Planar/Linear Supercapacitor with Outstanding Flexibility and Stability
Source: Nanomicro Lett. 2020 Jan 4;12:17. doi: 10.1007/s40820-019-0342-5 (PMC7770803; doi:10.1007/s40820-019-0342-5)
Supplement: Supplementary file 1 — Supplementary material 1 (PDF 1507 kb) [file 40820_2019_342_MOESM1_ESM.pdf]

Supporting Information for

Hierarchical Porous RGO/PEDOT/PANI Hybrid for Planar/Linear Supercapacitor with Outstanding Flexibility and Stability

Fuwei Liu[1, 2], Luoyuan Xie[1], Li Wang[1], Wei Chen[3], Wei Wei[4], Xian Chen[1], Shaojuan Luo[5], Lei Dong[6], Qilin Dai[7], Yang Huang[1, *], Lei Wang[1, *]

[1]Shenzhen Key Laboratory of Polymer Science and Technology, College of Materials Science and Engineering, Shenzhen University, Shenzhen 518060, People's Republic of China

[2]College of Physics and Electronic Engineering, Xinyang Normal University, Xinyang 464000, People's Republic of China

[3]Institute of Medical Engineering, School of Basic Medical Sciences, Xi'an Jiaotong University, Xi'an 710061, People's Republic of China

[4]Key Laboratory of Advanced Technologies of Materials (Ministry of Education), School of Materials Science and Engineering, Southwest Jiaotong University, Chengdu 610031, People's Republic of China

[5]School of Chemical Engineering and Light Industry, Guangdong University of Technology, Guangzhou 510006, People's Republic of China

[6]Department of Physics, Southern University of Science and Technology, Shenzhen 518055, People's Republic of China

[7]Department of Chemistry, Physics, and Atmospheric Sciences, Jackson State University, Jackson, Mississippi 39217, USA

*Corresponding authors. E-mail: y.huang@outlook.com (Yang Huang); wl@szu.edu.cn (Lei Wang)

S1 Experimental Section

S1.1 Materials

Analytical grade chemicals were used in this work as obtained. Aniline (Ani), vitamin C (Vc), perchloric acid ($HClO_4$, 71%), hydroiodic acid (HI, 57%), sulfuric acid (H_2SO_4, 98%), phosphoric acid (H_3PO_4, 85%) were all purchased from Sun Chemical Technology Co., Ltd. (Shanghai, China). Poly(vinyl alcohol) (PVA) was received from Sigma-Aldrich. PEDOT:PSS (Clevios PH1000) was obtained from Heraeus Company (Germany). Aqueous colloidal suspension of graphene oxide (GO) prepared via an improved Hummers method (1 layer thickness: 0.7~1.2 nm, 1 layer rate: >98.0 wt %) was provided by Suzhou TANFENG graphene Tech Co., Ltd. (Suzhou, China).

S1.2 Preparation of Porous GO/PEDOT:PSS Hybrid Film

Different amounts of GO and ascorbic acid (Vc) as referred to in the main text (0, 30, 60, 70, 80, 100 wt% for GO, and 0, 200, 400, 600, 680 wt% for Vc) were added to 3 mL PEDOT:PSS (5 mg mL^{-1}) solution to form a Vc/GO/PEDOT:PSS mixture. In order to achieve uniform dispersion, the mixture was sonicated in a bath ultra-sonicator for 1 h. Afterwards, the as-prepared solution was drop-cast on the cleaned glass substrates and dried at room temperature. When the films were fully shaped and dried, they were immersed into water to remove the pore-forming agent of Vc, and finally achieved the porous GO/ PEDOT:PSS hybrid films.

S1.3 Acid Treatments

To enhance electric conductivity of the porous GO/PEDOT:PSS hybrid film, the acid treatments were performed by soaking this porous film into concentrated $HClO_4$ and 57 wt% HI solution in succession, realizing the removal of PSS and reduction of GO, respectively. Then the treated film was rinsed with methanol and water alternately for several times and thus obtained a highly conductive and porous RGO/PEDOT hybrid framework.

S1.4 Preparation of Porous RGO/PEDOT/PANI Hybrid Film

A potentiostatic electrodeposition method was employed to grow PANI nanorods onto the porous RGO/PEDOT conductive framework in a three-electrode configuration. The porous RGO/PEDOT framework film was employed as working electrode, while the Pt mesh and saturated calomel electrode (SCE) were applied as counter electrode and reference electrode, respectively. The electrolyte was 25 mL 1.5 M pre-cooled $HClO_4$ solution containing a certain amount of Ani monomer (115 μL). PANI nanorods were electropolymerized onto the porous RGO/PEDOT with a constant voltage of 0.8 V vs. SCE for different periods (5, 10, 15, and 20 min). After electrodeposition of PANI, the obtained hybrid film was collected and washed using deionized water for several times and followed by drying at 60 °C in an oven for 12 h. For comparison, pure PANI nanorods were also synthesized in the absence of RGO/PEDOT via similar procedures.

S1.5 Preparation of Planar Type Solid-State Supercapacitor (SC)

The planar type solid-state SCs were fabricated by using porous RGO/PEDOT/PANI hybrid films as flexible electrodes and PVA/H_3PO_4 gel as solid electrolyte. For the preparation of PVA/H_3PO_4 electrolyte, 5 g of PVA and 3 mL of H_3PO_4 were added to 45 mL of deionized water, respectively, and the mixture was subsequently stirred under 95 °C until the formation of a clear solution. When the gel electrolyte was cooled down to room temperature, two equivalent pieces of RGO/PEDOT/PANI electrodes were immersed into the PVA/H_3PO_4 gel electrolyte and followed by a drying procedure under ambient condition. Then, to assemble a solid-state SC, these two electrodes were pressed together by sandwiching another layer of PVA/H_3PO_4 solid-electrolyte between them.

S1.6 Preparation of Linear Type Solid-State SC

A non-conductive cotton yarn was chosen as the substrate for linear SC. First, this cotton yarn was immersed into the as-prepared Vc/GO/PEDOT:PSS solution and followed by drying at room temperature. Second, the Vc/GO/PEDOT:PSS modified cotton yarn was immersed into water, $HClO_4$ and HI, sequentially, in order to remove Vc, PSS and reduce GO, respectively. After several alternate modifications of dip-coating and acidic treatments, the highly conductive RGO/PEDOT modified cotton yarn substrate was obtained. As for the electrodeposition of PANI, a conductive RGO/PEDOT modified yarn was directly used as working electrode, whereas Pt mesh and SCE were employed as counter electrode and reference electrode, respectively. The electrolyte and the electrodeposition method as used were the same with those of film electrode, the electrochemical deposition time was kept at 15 min. To fabricate a linear solid-state SC, two yarn electrodes with similar lengths were coated with a suitable amount of PVA/H_3PO_4 electrolyte and dried at room temperature for several hours. Then, they were parallelly assembled into a linear type solid-state SC by using a similar method like the planar SC.

S1.7 Characterizations

A Hitachi S-4700 field emission scanning electron microscopy (FE-SEM) was applied to investigate the surface and internal structure of different hybrid films. Transmission electron microscope (TEM) images of different micro/nanostructures were obtained on a JEOL-JEM F200 TEM operating at 200 kV. Raman spectra were recorded using a Renishaw inVia-Feflex confocal Raman microscope (UK) within a scan range from 500 to 2000 cm^{-1}. The molecular structures of samples were investigated using a Thermo Fisher Nicolet 6700 Fourier transform infrared spectrometer (FTIR) within the wavenumber range of 550~4000 cm^{-1}. X-ray diffraction (XRD) were performed using a Bruker D8 Advance X-ray diffractometer with Cu Kα radiation in the range 2θ = 5-60°. UV-Vis absorption spectra were collected by using an Evolution 220 UV-Visible spectrophotometer (Thermo Fisher, USA) in a wavelength range of 190-1100 nm. A Thermo Fisher ESCALAB 250Xi X-ray photoelectron spectroscopy (XPS) was used to measure the electron binding energy of the hybrid materials.

S1.8 Electrochemical Measurements

Electrochemical performance of the porous and hierarchical RGO/PEDOT/PANI hybrid film and its predecessors was investigated through a series of electrochemical measuring techniques including cyclic voltammogram (CV), galvanostatic charge-discharge (CD), and electrochemical impedance spectroscopy (EIS), which were performed on a CHI 660E electrochemical workstation (Shanghai CH Instruments Co., China). EIS was conducted in the frequency range of 10^5-10^{-2} Hz with an amplitude of 5 mV. Cycling stability was measured by using a CT2001A Land battery tester (Wuhan Landian Electronic Co., China).

The electrochemical performance of different hybrid film electrodes was investigated in a three-electrode configuration cell system while using 1 M H_2SO_4 as the electrolyte. The hybrid film electrodes, saturated calomel electrode and Pt mesh were used as working electrode, reference electrode and counter electrode, respectively. The specific capacitances (Cs) of different hybrid film electrodes were evaluated with Eq. S1:

$$C_s = It/m\Delta V \qquad \text{(S1)}$$

where I is constant discharge current (A), t presents discharge time (s), m and ΔV denote mass of hybrid film electrode (g) and potential window (V), respectively.

As for the planar type and linear type solid-state SC devices, the capacitive performances including specific capacitance (C_i: C_m, F g^{-1}/C_s, mF cm^{-2}), energy density (E_i: E_m, Wh kg^{-1}/E_s, mWh cm^{-2}) and power density (P_i: P_m, W kg^{-1}/P_s, mW cm^{-2}) were estimated according to Eqs. S2-S5:

$$C_m = 2It/m\Delta V \qquad \text{(S2)}$$

$$C_s = 2It/S\Delta V \qquad \text{(S3)}$$

$$E_i = \frac{1}{2}C_i\Delta V^2/3600 \qquad \text{(S4)}$$

$$P_i = \frac{E_i}{t} \times 3600 \qquad \text{(S5)}$$

where, I, t, ΔV, m and S, stands for discharge current, discharge time, discharge voltage range, mass and surface area of one single active electrode, respectively.

S2 Supplementary Figures and Disscussions

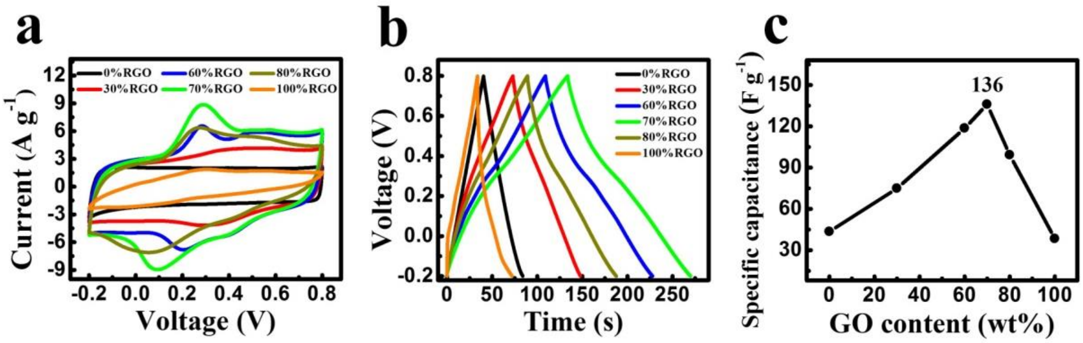

Fig. S1 Electrochemical properties of RGO/PEDOT: **a** CV curves of samples with different RGO content at 50 mV s^{-1}; **b** CD curves of samples with different RGO content at 1 A g^{-1}; **c** Specific capacitances of RGO/PEDOT with different RGO content obtained from CD curves

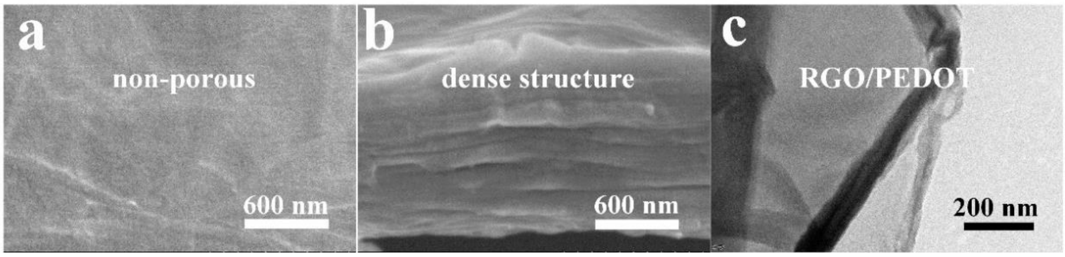

Fig. S2 a Surface and **b** cross-section SEM images of compact RGO/PEDOT. **c** TEM images of RGO/PEDOT

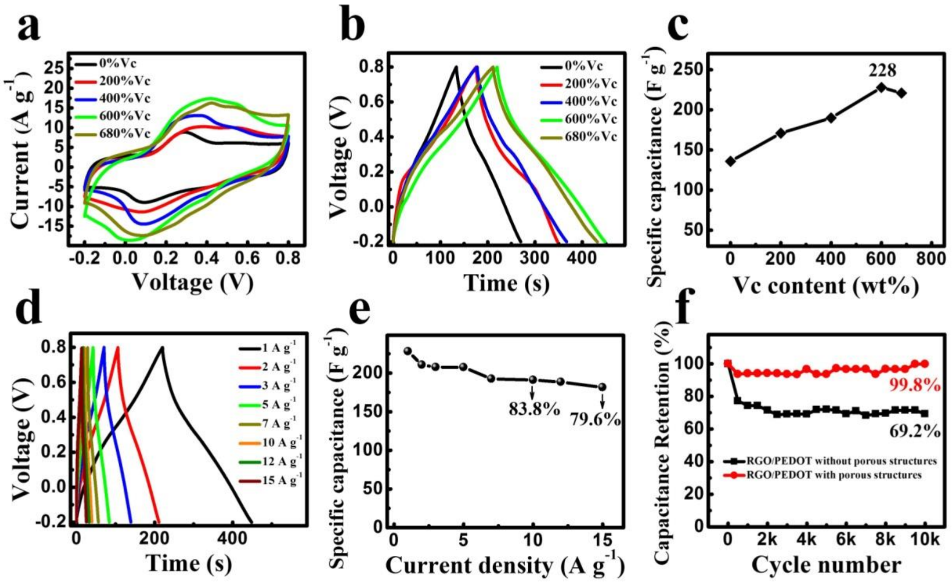

Fig. S3 Electrochemical properties of porous RGO/PEDOT (containing 70 wt% RGO) framework using different content of Vc as pore-forming agent: **a** CV curves of samples using different content of Vc at 50 mV s^{-1}; **b** CD curves of samples using different content of Vc at 1 A g^{-1}; **c** Specific capacitances of RGO/PEDOT using different content of Vc obtained from CD curves; **d** CD curves of samples using 600 wt% Vc at different current densities; **e** Specific capacitances of samples using 600 wt% Vc at different current densities; **f** Cycle stabilities of RGO/PEDOT (containing 70 wt% RGO) samples using 600 wt% and 0 wt% pore-forming agent of Vc after 10000 charging/discharging at 5 A g^{-1}

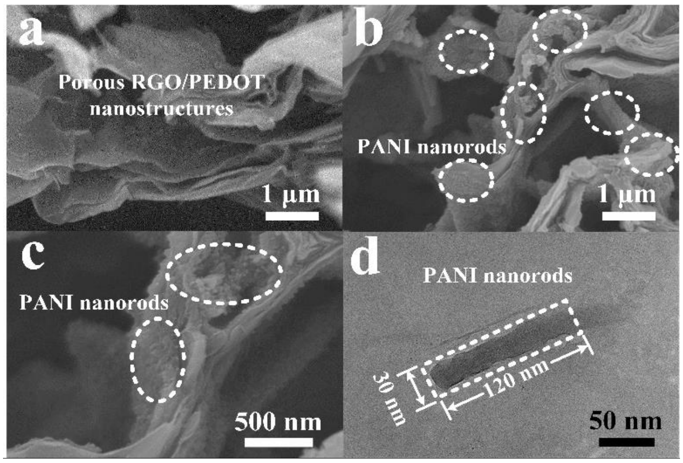

Fig. S4 Cross-section SEM images of **a** porous RGO/PEDOT and **b**, **c** porous RGO/PEDOT/PANI hybrid. **d** TEM image of PANI nanorod

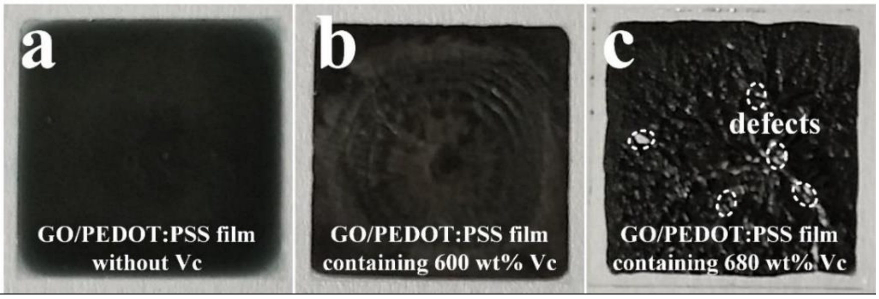

Fig. S5 Photographs of GO/PEDOT:PSS hybrid containing different Vc content: **a** without Vc; **b** 600 wt% Vc and **c** 680 wt% Vc

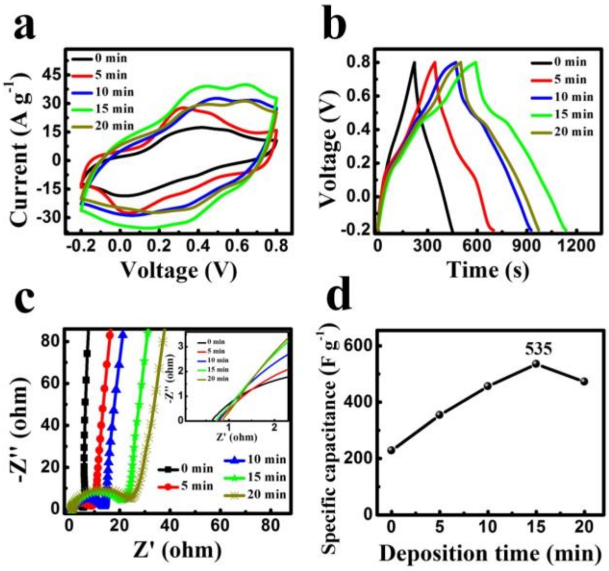

Fig. S6 Electrochemical properties of RGO/PEDOT/PANI hybrid: **a** CV curves of samples with different PANI electrodeposition durations at 50 mV s^{-1}; **b** CD curves of samples with different PANI electrodeposition durations at 1 A g^{-1}; **c** Nyquist plots of RGO/PEDOT/PANI hybrid; **d** Specific capacitances of RGO/PEDOT/PANI hybrids with different PANI electrodeposition durations obtained from CD curves

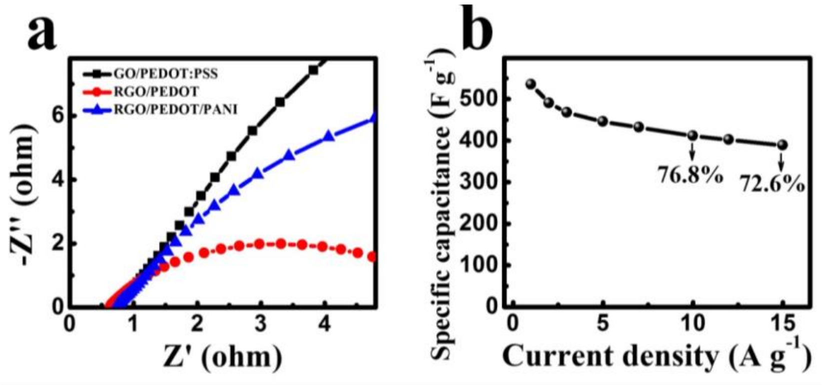

Fig. S7 a Nyquist plots of GO/PEDOT:PSS, RGO/PEDOT, and RGO/PEDOT/PANI hybrid; **b** Specific capacitances of RGO/PEDOT/PANI hybrid with different current densities obtained from CD curves

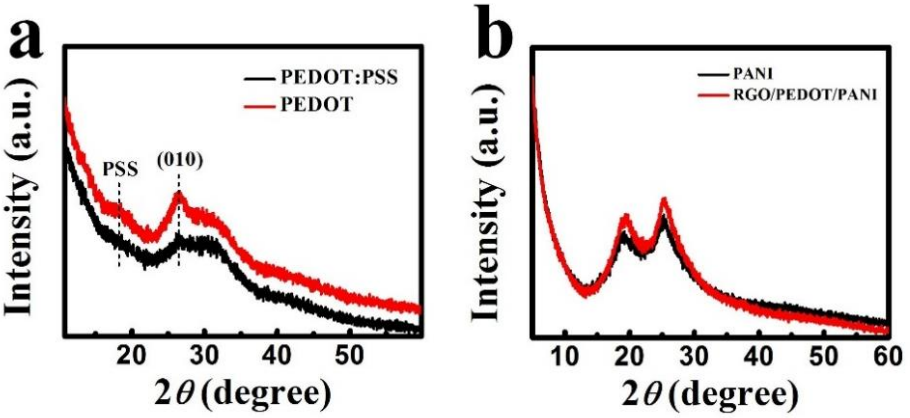

Fig. S8 XRD patterns of **a** PEDOT:PSS and PEDOT; **b** PANI film and RGO/PEDOT/PANI hybrid

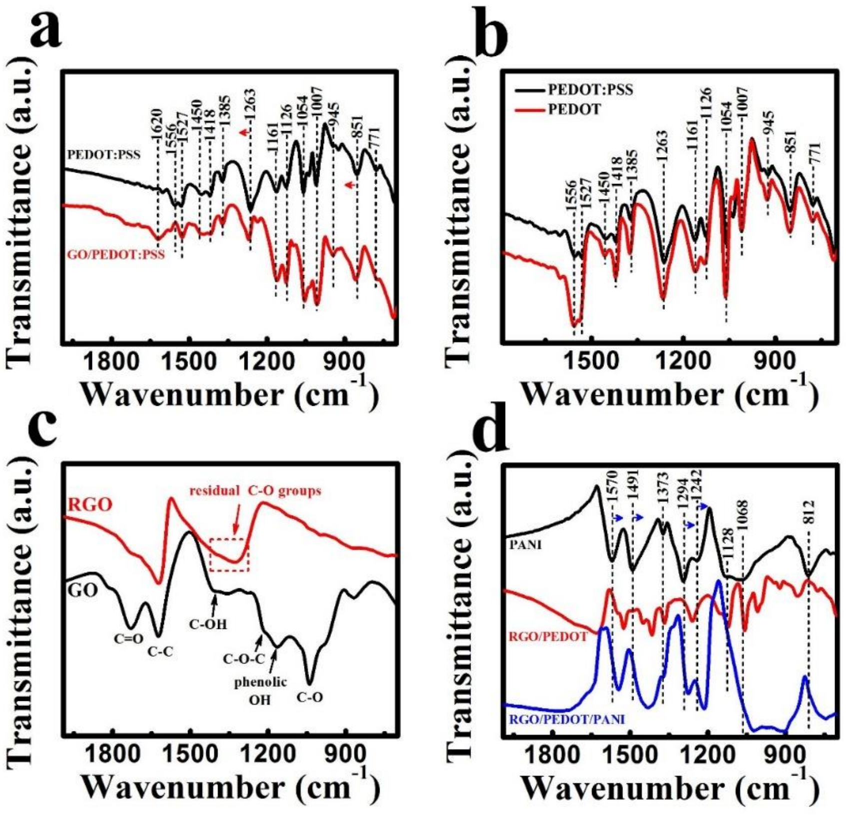

Fig. S9 FTIR spectra of **a** PEDOT:PSS and GO/PEDOT:PSS; **b** PEDOT:PSS and PEDOT; **c** GO and RGO; **d** PANI, RGO/PEDOT, and RGO/PEDOT/PANI

Figure S9 presents the FTIR spectra of the components in hybrid electrode after different treatments. As shown in Figure S9a, PEDOT:PSS shows typical

characteristic peaks of PEDOT and PSS: peaks at 1161 and 1126 cm^{-1} are related to S−O of PSS; peak at 1007 cm^{-1} is associated with S−phenyl bond of PSS; peaks at 1527 and 1450 cm^{-1} are attributed to C=C quinoidal structure of PEDOT; peaks at 1385, 1263, 1054 cm^{-1} are assigned to C−C, C−O−C bonds, and EDOT ring respectively; peaks at 945, 851, 771 cm^{-1} present C−S bonds of PEDOT.

It is worth noting that after compounding with GO, the peak associated with C−O−C bond at 1263 cm^{-1} slightly shifts to 1270 cm^{-1}, and the peak at 851 cm^{-1} related to C−S bond shifts to 857 cm^{-1}, which may be resulted from the strong interactions between GO and PEDOT:PSS molecular chains [S1−S2].

To identify the effect of acid treatment on molecular structure of PEDOT:PSS, we investigated the characteristic peaks of $HClO_4$ treated samples. The results were given in Figure S9b. Compared with PEDOT:PSS, PEDOT presents similar characteristic peaks with unobvious peak shifting after acid treatment. This may be owing to the limited effect of PSS removing on FTIR spectra after acid treatment. To further understand the structure changes induced by acid treatment, we also employed Raman, Uv-vis and XPS to characterize the corresponding variations in chemical and molecular structures as shown in the main text.

As well known, HI can eliminate the oxygen related groups of GO and change it from oxidation state to reduction one (RGO). Figure S9c presents the characteristic peaks of GO, including C=O stretching at 1730 cm^{-1}, skeletal vibration of deformed C−C at 1624 cm^{-1}, C−OH deformation at 1400 cm^{-1}, C−O (epoxy ring) stretching at 1230 cm^{-1}, phenolic OH stretching at 1165 cm^{-1}, and C−O stretching at 1040 cm^{-1}. After HI treatment, though most C=O, C−O, C−O−C, phenolic OH bonds (ranging from around 1300 cm^{-1} to 1040 cm^{-1}) were eliminated, some residual C−O related including C−OH chemical bonds (from 1400 cm^{-1} to around 1300 cm^{-1}) were observed in the RGO spectrum. All these changes in the reduction process of GO affect the structures of GO/PEDOT:PSS hybrid evidently and leads to a better conductivity as discussed in main text.

After electrodeposition, a series of typical peaks corresponding to the doped PANI appears in the hybrid. Additionally, some peaks shift to lower wavenumber compared with pure PANI (Fig. S9d). For example, peaks of C=C stretching vibration of quinonoid ring and benzenoid ring at 1570 cm^{-1} and 1491 cm^{-1} shift to 1545 and 1431 cm^{-1}, meanwhile peaks of stretching vibration of C−N bonds in secondary aromatic amines and C=N stretching at 1294 and 1242 cm^{-1} shift to 1276 and 1214 cm^{-1}. These results suggest that PANI nanorods were grown firmly on RGO/PEDOT with strong interfacial interactions, which is possibly attributed to the hydrogen bonding and π-π interaction.

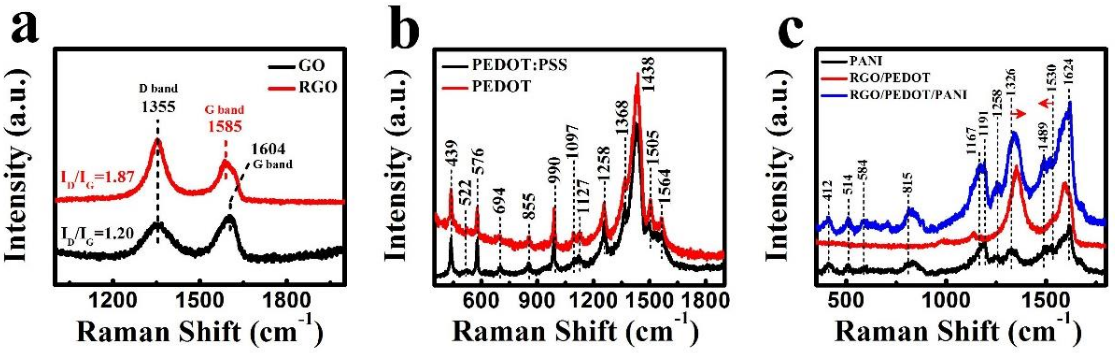

Fig. S10 Raman spectra of **a** GO and RGO; **b** PEDOT:PSS and PEDOT; **c** PANI, RGO/PEDOT, and RGO/PEDOT/PANI

Usually, D band is mainly associated with the disordered structure of graphene nanosheets such as vacancies, grain boundaries, and amorphous carbon species, while G band is ascribed to the E2g phonon of C sp^2 atoms in 2D lattice [S3−S4]. Defect density of the graphitic structure can be evaluated by calculating the intensity ratio of D and G band (I_D/I_G). Generally, an increase of I_D/I_G ratio means an increased defect density in graphitic structure, because presence of D band depends on the defects of graphene sheets. I_D/I_G is calculated to be 1.20 and 1.87 for GO and HI treated (RGO) samples (Fig. S10a), respectively, suggesting the reduction of GO causes an increase of structural defects.

As shown in Fig. S10b, PEDOT:PSS presents no obvious Raman shift before and after acid treatments, which is owing to the limited effect of PSS on Raman spectra when it is removed from PEDOT after acid treatment. As shown in the spectra, most of the bands are related to PEDOT structure: 1564 cm^{-1} and 1505 cm^{-1} are assigned to the asymmetric stretching $C_\alpha=C_\beta$, 1438 cm^{-1} to symmetric stretching $C_\alpha=C_\beta(-O)$ on five-membered ring, 1368 cm^{-1} to $C_\beta-C_\beta$ stretching, 1258 cm^{-1} to $C_\alpha-C_\alpha$ inter-ring stretching, 1127 cm^{-1} to C−C inter-ring bending variation, 1097 cm^{-1} to C−O−C deformation, 990 cm^{-1} and 576 cm^{-1} to oxyethylene ring deformation, 694 cm^{-1} to symmetric C−S−C deformation, and 439 cm^{-1} to SO_2 bending; whereas only the peak at 522 cm^{-1} is related to PSS part [S5−S6]. Thus, Raman spectrum of the sample measured after acid treatment remains almost the same with pristine PEDOT:PSS, though some peak intensity varies in some extent, for instance, peaks ascribed to the asymmetric stretching $C_\alpha=C_\beta$ (1564 cm^{-1} and 1505 cm^{-1}), $C_\alpha-C_\alpha$ inter-ring stretching (1258 cm^{-1}), and oxyethylene ring deformation (990 cm^{-1}) all increase. Therefore, we infer that there is some conformational and compositional changes in structure of PEDOT:PSS after acid treatment, which is further investigated by other characterizations, such as Uv-vis and XPS.

The Raman spectra of RGO/PEDOT/PANI is much more different from its predecessors. Some new peaks belong to PANI emerge as shown in Table S3. It is worth noting that some peaks slightly shift when compared with pure PANI. For example, the $C-N^+$ vibration (quinoid ring in pure PANI) peak at 1326 cm^{-1} slightly shifts to higher wavenumber direction (1345 cm^{-1}, Fig. S10c), while N−H stretching vibration peak at 1530 cm^{-1} shifts to lower wavenumber direction (1521 cm^{-1}) with much higher intensity.

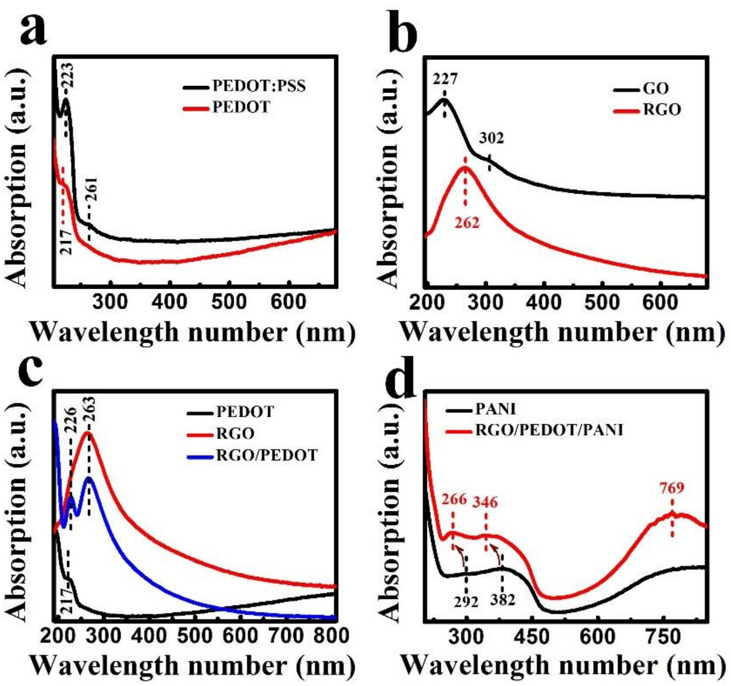

Fig. S11 Uv-vis spectra of **a** PEDOT:PSS and PEDOT; **b** GO and RGO; **c** PEDOT, RGO, and RGO/PEDOT; **d** PANI and RGO/PEDOT/PANI

The spectrum of GO/PEDOT:PSS shows absorbance peaks at 226, 251 and 302 nm, respectively, which corresponds to the aromatic ring of PSS group in pristine PEDOT:PSS and/or GO nanostructures as shown in FigS. 4d and S11a [S7]. After acid treatments, peaks at 251 and 302 nm disappear, which is attributed to the decrease of PSS group in PEDOT and reduction of GO (Fig. S11a, b). Meanwhile, there is a new absorbance peak appears at 263 nm corresponding to the π–π* transitions of C−C bonds in aromatic ring, which is the result of GO reduction (Figs. 4d and S11b) [S8]. In fact, compared with the Raman spectra results, corresponding Uv-vis spectrum of each component of this GO/PEDOT framework could indicate its obvious structural changes after respective acid treatments. For example, PEDOT spectrum shows only a weak shoulder peak at 217 nm after $HClO_4$ treatment (Fig. S11a), whereas GO exhibits a single peak at 262 nm after treating with HI (Fig. S11b). Obviously, the absorption spectra of RGO/PEDOT framework are not just a simple combination of each component, since the absorption peaks present at different wavelength or with a slight shifting (Fig. S11c), which suggests the strong interaction between different components.

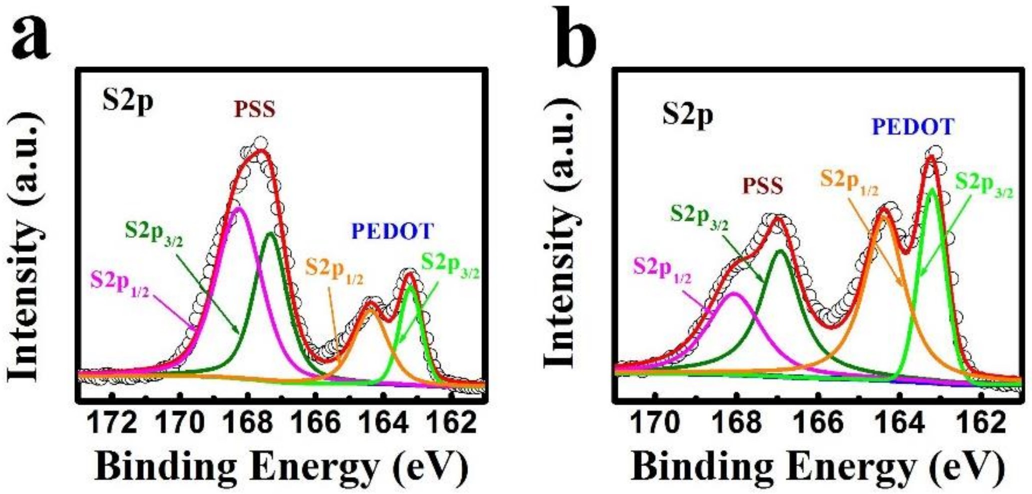

Fig. S12 XPS spectra of PEDOT:PSS and PEDOT: **a** S2p spectra of PEDOT:PSS; **b** S2p spectra of PEDOT

As shown in Fig. S12, S 2p peaks in pure PEDOT:PSS change tremendously after identical acid treatments. Because the binding energies of sulfur atoms in the thiophene unit of PEDOT are different with that of sulfonate group in PSS, S2p peaks can be resolved into two distinct types of sulfur atoms. The broad peak at higher binding energy is attributed to the overlap of $S2p_{3/2}$ and $S2p_{1/2}$ peaks (167.3 and 168.3 eV) of S atoms in PSS, while the doublet $S2p_{3/2}$ and $S2p_{1/2}$ peaks (163.2 and 164.4 eV) at lower binding energy arise from the S atoms in PEDOT [S9−S11]. The higher S 2p binding energy of PSS is owing to the spontaneous attachment of electronegative oxygen on sulfonate moiety. Specifically, the ratio of $S2p_{3/2}$ peak integral area of PSS to that of PEDOT can be used to quantitatively estimate the compositional changes of PEDOT:PSS [S9]. After acid treatment, the ratio of PSS to PEDOT was altered from 2.54 to 1.42, a direct proof of PSS's removal from PEDOT:PSS.

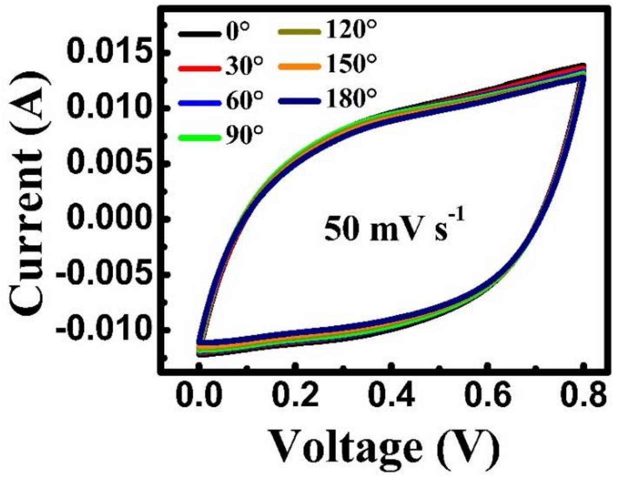

Fig. S13 CV curves of the flexible solid-state SC at different bending angles

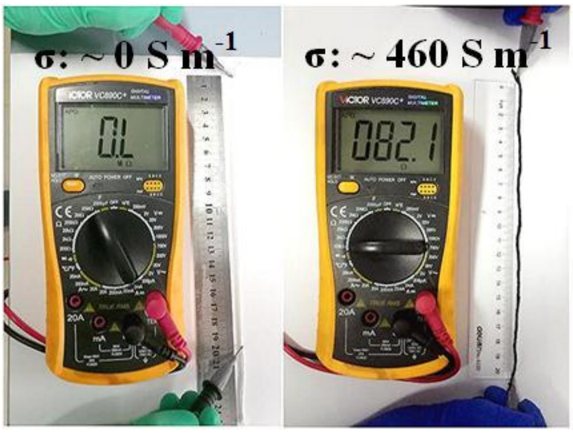

Fig. S14 Conductivities of pristine cotton yarn (left) and RGO/PEDOT modified cotton yarn (right)

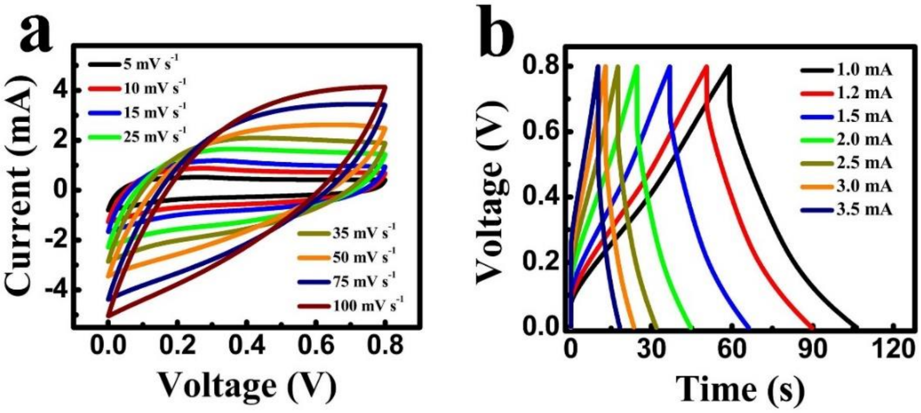

Fig. S15 a CV curves of the linear SC device based on RGO/PEDOT/PANI hybrids at different scan rates. **b** CD curves of the linear device at different current densities

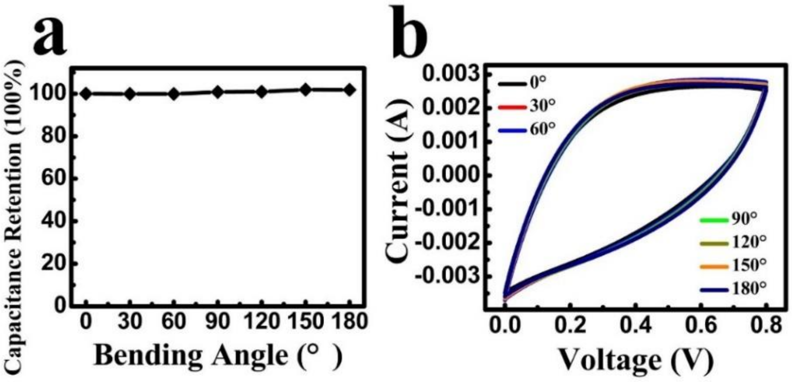

Fig. S16 a Capacitance retention at different bending angles. **b** CV curves of the linear SC device at different bending angles

Table S1 Electrochemical performance of the reported RGO-, PEDOT-, and PANI-based hybrid electrodes

Materials	Fabrication method	Electrolyte	Potential window	Specific capacitance	Cycle life retention	Year/Refs.
CNFs@PPy@RGO	Carbonization and electrochemical deposition	3 M KCl	0 ~ 1 V	336.2 F g^{-1} at 2 mV s^{-1}	98% (2500)	2018/[S12]
RGO/PANI	In situ one-pot synthesis route	1 M H_2SO_4	−0.2 ~ 0.7 V	524.4 F g^{-1} at 0.5 A g^{-1}	81.1% (2000)	2017/[S13]
RGO/PANI/UMTS	In situ polymerization and hydrothermal method	1 M HCl	0 ~ 0.6 V	464 F g^{-1} at 0.62 A g^{-1}	86.5% (2000)	2016/[S14]
RGO/PANI	In situ polymerization	3 M NaOH	−0.2 ~ 0.6 V	596 F g^{-1} at 1 A g^{-1}	>85% (1000)	2017/[S15]
MnO_2-RGO	Hydrothermal method	6 M KOH	0 ~ 0.45 V	486.48 F g^{-1} at 0.85 A g^{-1}	74.8% (2000)	2017/[S16]
3D-RGO/PANI	Layer-by-layer assembly in situ polymerization	1 M H_2SO_4	0 ~ 0.8 V	438.8 F g^{-1} at 0.5 A g^{-1}	76.5% (2000)	2017/[S17]
RGO/PANI-HS	Chemical polymerization and Hydrothermal method	1 M H_2SO_4	0 ~ 0.8 V	529 F g^{-1} at 0.5 A g^{-1}	85% (1000)	2016/[S18]
PEDOT/MnO_2	Thermal treatment and chemical vapor polymerization	1 M H_2SO_4	−0.2 ~ 0.8 V	321.4 F g^{-1} at 0.5 A g^{-1}	>90% (4000)	2015/[S19]
PEDOT–PSS–PANI–MnO_2	Interfacial polymerization and electrodeposition	0.1 M Na_2SO_4	0 ~ 1 V	61.5 F g^{-1} ----	68% (500)	2008/[S20]
MnO_2/CNT/CP	Sonochemical coprecipitation	1 M Na_2SO_4	0 ~ 1 V	427 F g^{-1} at 1 mA cm^{-2}	99% (1000)	2010/[S21]
graphene/MnO_2/PEDOT:PSS	Solution-based coating process and wrapping method	0.5 M Na_2SO_4	0 ~ 0.85 V	380 F g^{-1} at 0.5 mA cm^{-2}	~95% (3000)	2011/[S22]
Cr_2O_3/GO/PANI	One-step chronoamperometry method	0.5 M Na_2SO_4	−0.2 ~ 0.8 V	525 F g^{-1} at 5 A g^{-1}	84% (4000)	2018/[S23]
3-D graphene/PANI nanorod	Hydrothermal treatment in-situ synthesis	1 M Na_2SO_4	0 ~ 1 V	352 F g^{-1} at 10 mV s^{-1}	----	2017/[S24]
PANI/GO	Chemical polymerization and a simple solution-mixing method	6 M KOH	0 ~ 0.4 V	475 F g^{-1} at 5 A g^{-1}	90% (2000)	2018/[S25]
PANI/UMTS	Grafting polymerization	1 M HCl	0 ~ 0.6 V	422 F g^{-1} at 0.7 A g^{-1}	100% (500)	2015/[S26]
N-doped graphene/PANI hydrogels	Hydrothermal process	2 M H_2SO_4	−0.2 ~ 0.6 V	514.3 F g^{-1} at 1 A g^{-1}	84.7% (1000)	2018/[S27]
GO@MSNs/PANI	Wrapping and interfacial polymerization	1 M H_2SO_4	−0.2 ~ 0.8 V	412 F g^{-1} at 1 A g^{-1}	----	2018/[S28]
porous RGO/PEDOT/PANI	Acid treatment and electrodeposition	1 M H_2SO_4	−0.2 ~ 0.8 V	535 F g^{-1} at 1 A g^{-1}	99%(10000)	**this work**

Table S2 Assignments of the main characteristic FTIR peaks in RGO/PEDOT/PANI free-standing film [S29, S30]

Wavenumber (cm^{-1})	Assignments
1545	C=C stretching of quinoid ring
1431	C=C stretching of benzene ring
1276	stretching vibration of C−N bonds in secondary aromatic amines
1214	C=N stretching
1022	in-plane bending vibration of C−H

745	Out-of-plane bending vibration of C−H

Table S3 Assignments of the main characteristic Raman bands for RGO/PEDOT/PANI free-standing film [S31, S32]

Raman bands (cm^{-1})	Assignments
412, 514	out-of-plane PANI ring band
584	benzenod ring deformation
815	out-of plane vibrations of aromatic rings
1167	C–H vibration
1191	C–H bending vibration of quinoid/benzenoid ring
1258	C–N stretching vibration
1345	C–N^+ vibration of quinoid ring
1489	C=N of di-imine units
1521	N–H stretching vibration
1599	C–C stretching of semiquinoid ring
1624	vibration of delocalized polarons in the extended polymeric conformation

Supplementary References

[S1] F. Ely, A. Matsumoto, B. Zoetebier, V. S. Peressinotto, M. K. Hirata, D. A. d. Sousa, R. Maciel, Handheld and automated ultrasonic spray deposition of conductive PEDOT:PSS films and their application in AC EL devices. Org. Electron. **15**(5), 1062−1070 (2014). https://doi.org/10.1016/j.orgel.2014.02.022

[S2] X. Zhao, H. Lin, J. Li, L. Xin, C. Liu, J. Li, Low-cost preparation of a conductive and catalytic graphene film from chemical reduction with AlI3. Carbon **50**(10), 3497−3502 (2012). https://doi.org/10.1016/j.carbon.2012.03.017

[S3] K. Dagci, M. Alanyalioglu, Preparation of free-standing and flexible graphene/Ag nanoparticles/poly(pyronin Y) hybrid paper electrode for amperometric determination of nitrite. ACS Appl. Mater. Interfaces **8**(4), 2713–2722 (2016). https://doi.org/10.1021/acsami.5b10973

[S4] M. Alanyalıoğlu, J. J. Segura, J. Oró-Solè, N. Casañ-Pastor, The synthesis of graphene sheets with controlled thickness and order using surfactant-assisted electrochemical processes. Carbon **50**(1), 142–152 (2012). https://doi.org/10.1016/j.carbon.2011.07.064

[S5] A. A. Farah, S. A. Rutledge, A. Schaarschmidt, R. Lai, J. P. Freedman, A. S. Helmy, Conductivity enhancement of poly(3,4-ethylenedioxythiophene) poly(styrenesulfonate) films post-spincasting. J. Appl. Phys. **112**(11), 113709 (2012). http://dx.doi.org/10.1063/1.4768265

[S6] A. Schaarschmidt, A. A. Farah, A. Aby, A. S. Helmy, Influence of nonadiabatic annealing on the morphology and molecular structure of PEDOT-

PSS films. J. Phys. Chem. B **113**(28), 9352−9355 (2009). https://doi.org/10.1021/jp904147v

[S7] P. C. Mahakul, K. Sa, B. Das, B. V. R. S. Subramaniam, S. Saha, B. Moharana, J. Raiguru, S. Dash, J. Mukherjee, P. Mahanandia, Preparation and characterization of PEDOT:PSS/reduced graphene oxide–carbon nanotubes hybrid composites for transparent electrode applications. J. Mater. Sci. **52**(10), 5696–5707 (2017). https://doi.org/10.1007/s10853-017-0806-2

[S8] D. Li, Y. Li, Y. Feng, W. Hu, W. Feng, Hierarchical graphene oxide/polyaniline nanocomposites prepared by interfacial electrochemical polymerization for flexible solid-state supercapacitors. J. Mater. Chem. A **3**, 2135–2143 (2015). https://doi.org/10.1039/C4TA05643D

[S9] E. Jin Bae, Y. Hun Kang, K. -S. Jang, S. Yun Cho, Enhancement of thermoelectric properties of PEDOT:PSS and tellurium-PEDOT:PSS hybrid composites by simple chemical treatment. Sci. Rep. **6**, 18805 (2016). https://doi.org/10.1038/srep18805

[S10] H. Yan, H. Okuzaki, Effect of solvent on PEDOT/PSS nanometer-scaled thin films: XPS and STEM/AFM studies. Synthetic Met. **159**(21–22), 2225–2228 (2009). https://doi.org/10.1016/j.synthmet.2009.07.032

[S11] H. Ling, J. Lu, S. Phua, H. Liu, L. Liu, Y. Huang, D. Mandler, P. S. Lee, X. Lu, One-pot sequential electrochemical deposition of multilayer poly(3,4-ethylenedioxythiophene):poly(4-styrenesulfonic acid)/tungsten trioxide hybrid films and their enhanced electrochromic properties. J. Mater. Chem. A **2**, 2708–2717 (2014). https://doi.org/10.1039/C3TA14781A

[S12] L. Chen, L. Chen, Q. Ai, D. Li, P. Si, J. Feng, L. Zhang, Y. Li, J. Lou, L. Ci, Flexible all-solid-state supercapacitors based on freestanding, binder-free carbon nanofibers@polypyrrole@graphene film. Chem. Eng. J. **334**, 184–190 (2018). https://doi.org/10.1016/j.cej.2017.10.038

[S13] N. Chen, Y. Ren, P. Kong, L.Tan, H. Feng, Y. Luo, In situ one-pot preparation of reduced graphene oxide/polyaniline composite for high-performance electrochemical capacitors. Appl. Surf. Sci. **392**, 71–79 (2017). https://doi.org/10.1016/j.apsusc.2016.07.168

[S14] H. Wang, L. Ma, M. Gan, T. Zhou, X. Sun, W. Dai, H. Wang, S. Wang, Design and assembly of reduced graphene oxide/polyaniline/urchin-like mesoporous TiO_2 spheres ternary composite and its application in supercapacitors. Compos. Part B-Eng. **92**, 405–412 (2016). https://doi.org/10.1016/j.compositesb.2016.02.047

[S15] L. Tang, Z.Yang, F. Duan, M. Chen, Fabrication of graphene sheets/polyaniline nanofibers composite for enhanced supercapacitor properties. Colloids Surf. A **520**, 184–192 (2017). https://doi.org/10.1016/j.colsurfa.2017.01.083

[S16] N. Parveen, S. A. Ansari, M. O. Ansari, M. H. Cho, Manganese dioxide nanorods intercalated reduced graphene oxide nanocomposite toward high performance electrochemical supercapacitive electrode materials. J. Colloid Interface Sci. **506**, 613–619 (2017). https://doi.org/10.1016/j.jcis.2017.07.087

[S17] X. Hong, B. Zhang, E. Murphy, J. Zou, F. Kim, Three-dimensional reduced graphene oxide/polyaniline nanocomposite film prepared by diffusion driven layer-by-layer assembly for high-performance supercapacitors. J. Power Sources **343**, 60–66 (2017). https://doi.org/10.1016/j.jpowsour.2017.01.034

[S18] W. Dai, L. Ma, M. Gan, S. Wang, X. Sun, H. Wang, H. Wang, T. Zhou, Fabrication of sandwich nanostructure graphene/polyaniline hollow spheres composite and its applications as electrode materials for supercapacitor. Mater. Res. Bull. **76**, 344–352 (2016). https://doi.org/10.1016/j.materresbull.2015.12.045

[S19] Y. Yang, W. Yuan, S. Li, X. Yang, J. Xu, Y. Jiang, Manganese dioxide nanoparticle enrichment in porous conducting polymer as high performance supercapacitor electrode materials. Electrochim. Acta **165**, 323–329 (2015). https://doi.org/10.1016/j.electacta.2015.03.052

[S20] F.-J. Liu, Electrodeposition of manganese dioxide in three-dimensional poly(3,4-ethylenedioxythiophene)–poly(styrene sulfonic acid)–polyaniline for supercapacitor. J. Power Sources **182**(1), 383–388 (2008). https://doi.org/10.1016/j.jpowsour.2008.04.008

[S21] Y. Hou, Y. Cheng, T. Hobson, J. Liu, Design and synthesis of hierarchical MnO_2 nanospheres/carbon nanotubes/conducting polymer ternary composite for high performance electrochemical electrodes. Nano Lett. **10**(7), 2727–2733 (2010). https://doi.org/10.1021/nl101723g

[S22] G. Yu, L. Hu, N. Liu, H. Wang, M. Vosgueritchian, Y. Yang, Y. Cui, Z. Bao, Enhancing the supercapacitor performance of graphene/MnO_2 nanostructured electrodes by conductive wrapping. Nano Lett. **11**(10), 4438–4442 (2011). https://doi.org/10.1021/nl2026635

[S23] P. Asen, S. Shahrokhian, A. I. zad, Ternary nanostructures of Cr_2O_3/graphene oxide/conducting polymers for supercapacitor application. J. Electroanal. Chem. **823**, 505–516 (2018). https://doi.org/10.1016/j.jelechem.2018.06.048

[S24] T. Zhao, X. Ji, P. Bi, W. Jin, C. Xiong et al., In situ synthesis of interlinked three-dimensional graphene foam/polyaniline nanorod supercapacitor. Electrochim. Acta **230**, 342–349 (2017). https://doi.org/10.1016/j.electacta.2017.02.021

[S25] T.-W. Chang, L.-Y. Lin, P.-W. Peng, Y.X. Zhang, Y.-Y. Huang, Enhanced electrocapacitive performance for the supercapacitor with tube-like polyaniline and graphene oxide composites. Electrochim. Acta **259**, 348–354 (2018). https://doi.org/10.1016/j.electacta.2017.10.195

[S26] H. Wang, L. Ma, M. Gan, T. Zhou, X. Sun, W. Dai, H. Wang, S. Wang, Fabrication of polyaniline/urchin-like mesoporous TiO_2 spheres nanocomposite and its application in supercapacitors. Electrochim. Acta **163**, 232–237 (2015). https://doi.org/10.1016/j.electacta.2015.02.088

[S27] Y. Zou, Z. Zhang, W. Zhong, W. Yang, Hydrothermal direct synthesis of polyaniline, graphene/polyaniline and N-doped graphene/polyaniline hydrogels for high performance flexible supercapacitors. J. Mater. Chem. A **6**, 9245–9256 (2018). https://doi.org/10.1039/C8TA01366G

[S28] M. Javed, S.M. Abbas, M. Siddiq, D. Han, L. Niu, Mesoporous silica wrapped with graphene oxide-conducting PANI nanowires as a novel hybrid electrode for supercapacitor. J. Phys. Chem. Solids **113**, 220–228 (2018). https://doi.org/10.1016/j.jpcs.2017.10.037

[S29] J. Li, Y. Ren, Z. Ren, S. Wang, Y. Qiu, J. Yu, Aligned polyaniline nanowires grown on the internal surface of macroporous carbon for supercapacitors. J. Mater. Chem. A **3**, 23307–23315 (2015). https://doi.org/10.1039/C5TA05381A

[S30] T. Yu, P. Zhu, Y. Xiong, H. Chen, S. Kang, H. Luo, S. Guan, Synthesis of microspherical polyaniline/graphene composites and their application in supercapacitors. Electrochim. Acta **222**(20), 12–19 (2016). https://doi.org/10.1016/j.electacta.2016.11.033

[S31] D. Liu, H. Wang, P. Du, W. Wei, Q. Wang, P. Liu, Flexible and robust reduced graphene oxide/carbon nanoparticles/polyaniline (RGO/CNs/PANI) composite films: Excellent candidates as free-standing electrodes for high-performance supercapacitors. Electrochim. Acta **259**(1), 161–169 (2018). https://doi.org/10.1016/j.electacta.2017.10.165

[S32] X. Li, C. Zhang, S. Xin, Z. Yang, Y. Li, D. Zhang, P. Yao, Facile synthesis of MoS_2/reduced graphene oxide@polyaniline for high-performance supercapacitors. ACS Appl. Mater. Interfaces **8**(33), 21373–21380 (2016). https://doi.org/10.1021/acsami.6b06762
